# Global, regional, and national burden of decubitus ulcers, 1990–2021: analysis of the current situation, multidimensional analysis, and trend forecasting for the global burden of disease study 2021

**DOI:** 10.3389/fmed.2025.1588032

**Published:** 2025-07-02

**Authors:** Zhaoyi Jing, Qingyu Song, Bingbing Wang, Xiao Ding, Wei Yan, Xianghua Qi

**Affiliations:** ^1^The First School of Clinical Medicine, Shandong University of Traditional Chinese Medicine, Jinan, China; ^2^The Seventh Clinical College, Shanghai University of Traditional Chinese Medicine, Shanghai, China; ^3^Department of Neurology II, Shandong University of Traditional Chinese Medicine Affiliated Hospital, Jinan, China

**Keywords:** global disease burden, decubitus ulcers, health inequality analysis, decomposition analysis, frontier analysis

## Abstract

**Background:**

This study aimed to deepen the understanding and assessment of the global burden of decubitus ulcers to provide evidence for policy making and resource allocation.

**Methods:**

Using the standardized methodology of the 2021 Global Burden of Disease study, the disease burden of decubitus ulcers was analyzed at the global, regional, and national levels, with a focus on age and gender factors. The study also included health inequality analysis, decomposition analysis, and frontier analysis. The disease burden of decubitus ulcers for the year 2035 was projected.

**Results:**

From 1900 to 2021, the incidence, prevalence, and mortality of decubitus ulcers increased continuously. When analyzed by Socio-demographic Index (SDI), incidence decreased slightly in high-SDI regions but increased in all other regions. India was found to bear the heaviest burden of mortality and disability-adjusted life-years (DALYs), while the United States had the highest incidence and prevalence. Health inequality persisted, with the level of inequality in DALYs showing a greater increase compared to 1990. Decomposition analysis indicated that population aging and population growth remained the primary drivers of the increased burden of decubitus ulcers, with some regional variations. Frontier analysis revealed that countries positioned on the disease burden frontier were primarily located in middle-high and high SDI regions.

**Conclusion:**

The burden of decubitus ulcers remains substantial globally, with marked disparities across regions and nations. A disproportionately high share of this burden affects older adult populations. Implementation of targeted health policies is warranted to mitigate the global burden of decubitus ulcers.

## 1 Introduction

Decubitus ulcers (DUs) are localized damage to the skin and/or underlying soft tissue, typically occurring over bony prominences (1). This condition results from prolonged pressure that compromises blood flow, leading to tissue ischemia, skin breakdown, ulceration, and, in severe cases, necrosis (2). DUs predominantly affect individuals with limited mobility, such as those who are bedridden or wheelchair-bound, particularly when sensory perception or circulatory function is impaired (3). Despite significant advancements in medical understanding and the development of innovative prevention, diagnostic, and therapeutic strategies, pressure ulcers persist as a widespread and debilitating health issue (4). They impose a substantial burden not only on affected individuals but also on healthcare systems and society at large, due to their associated economic and resource costs.

Global population restructuring, driven by social changes and economic development, has profound implications for healthcare systems (5). Between 1990 and 2019, the global population increased by 43%, necessitating adaptation of national healthcare infrastructure to accommodate demographic pressures. However, in recent years, the specific impact of global population growth on the burden of DUs has not been systematically studied. In addition, the global outbreak of the novel coronavirus has significantly increased the number of bedridden patients which has had a significant impact on the prevalence of DUs (6). This research gap impedes evidence-based policy formulation by the World Health Organization and national governments seeking to address population-driven healthcare demands. Comprehensive analysis of global DUs epidemiology and future projections would provide critical insights for preventive strategies. Currently, no study reports longitudinal data on mortality, prevalence, incidence, and disability-adjusted life years (DALYs) attributable to DUs across all countries. Given the substantial physical, societal, and public health impacts of DUs, understanding their epidemiology is essential for optimizing resource allocation toward prevention and disease management.

The Global Burden of Disease 2021 (GBD 2021) study encompasses 204 countries and regions, employing enhanced statistical methodologies to ensure data accuracy and reliability. This investigation systematically characterized the 2021 disease burden of DUs using the GBD modeling framework. Data were processed and analyzed at global, regional, and national levels to evaluate temporal trends and epidemiological patterns. Age-specific distributions and socioeconomic determinants of DUs were examined. Cross-national inequality analysis and frontier analysis of DUs burden were conducted for the first time. The impacts of population aging, demographic growth, and epidemiological transitions on DUs burden were quantified through decomposition analysis. Projections of disease burden trends were extended to 2035. This comprehensive assessment enables monitoring of DUs progression, identification of health disparities, and formulation of targeted policies to enhance global and regional health outcomes.

## 2 Materials and methods

### 2.1 Data acquisition and research direction

This cross-sectional study utilized the Global Health Data Exchange (GHDx) query tool to access standardized data on DUs, including case definitions and incidence rates. All methods were performed in accordance with relevant guidelines and regulations, with ethical approval obtained from the Institutional Review Board of the Affiliated Hospital of Shandong University of Traditional Chinese Medicine. As the study involved secondary analysis of anonymized, non-identifiable data, informed consent was waived.

Data were derived from the Global Burden of Disease 2021 study, which features enhanced epidemiological modeling through optimized statistical approaches including Bayesian hierarchical models and spatiotemporal smoothing techniques. Key methodological advancements included explicit adjustment for COVID-19 pandemic impacts, expanded health indicators, and incorporation of broader data sources, establishing GBD 2021 as the most comprehensive global epidemiological resource. We extracted age-standardized epidemiological metrics for DU incidence, prevalence, and DALYs at global, regional, and national levels, including 95% uncertainty intervals (UI) from the GBD Results Tool.^1^

### 2.2 Research dimensions

This study employed the Social Demographic Index (SDI) to analyze the association between DUs and the socio-economic development level of various countries or regions. The SDI integrates factors such as the average years of education for the population aged 15 and above, the total fertility rate of women under 25, and the lagged effect of income distribution. Countries and regions were categorized into five development levels (Low, Low-Middle, Middle, High-Middle, and High), with values ranging from 0 to 1, where closer to 1 signifies higher socioeconomic development status. Countries were stratified into five socioeconomic development strata based on SDI quintiles (range: 0–1; higher values indicate greater development): Low SDI (<0.45), Low-middle SDI (0.45–0.60), Middle SDI (0.61–0.74), High-middle SDI (0.75–0.89), and High SDI (≥0.90). Furthermore, the study analyzed disease burdens from global, regional, and national perspectives, exploring disease disparities among different age groups and describing trends over time.

## 3 Statistical analysis

Age-standardized rates (ASR) and their corresponding 95% uncertainty intervals (UI) were utilized to assess the rates of incidence, prevalence, mortality and DALYs.

### 3.1 Joinpoint regression model

Joinpoint regression modeling was applied to identify optimal trend-fitting points and significant temporal inflection points in disease burden (7). The annual percentage change (APC) quantified segment-specific trends, while the average annual percentage change (AAPC) characterized overall trends from 1990 to 2021. Additionally, Spearman correlation analysis evaluated the association between ASR and SDI, including R index and *p*-value, while Pearson coefficient measured the correlation between Estimated Annual Percentage Change (EAPC) and SDI, ASR. The Joinpoint Regression Program and R software were used to perform data analysis. Statistical significance was defined as a *p* value of less than 0.05.

### 3.2 Health inequality analysis

Health inequality analysis is used to assess variations in health status across populations, aiming to understand the correlations between characteristics such as socioeconomic status, geographic location, gender, and age, as well as their impact on health outcomes (8). To investigate the socioeconomic disparity distribution of DUs among different countries and regions globally, the study employed two standard indicators: the slope index of inequality (SII) and the concentration index (CI).

### 3.3 Decomposition analysis

Decomposition analysis was used to visually demonstrate the role of the three factors driving changes in DALYs between 1990 and 2019 (i.e., aging, population, and epidemiology) (9, 10). Decomposition analysis was conducted to elucidate the main factors driving changes in the disease burden of DUs between 1990 and 2021. Population expansion, linked to changes in overall population size, influenced disease burden outcomes. Specifically, even when incidence and mortality rates remain constant, rapid population growth can exacerbate disease burden. Population aging represents a phenomenon in which an increasing proportion of older adult individuals within a population may lead to a greater burden of chronic and non-communicable diseases. Epidemiological transition refers to shifts in disease incidence or mortality patterns, reflecting advances in medical technology and public health initiatives.

### 3.4 Frontier analysis

The Free Disposal Hull (FDH) method combined with Data Envelopment Analysis (DEA) is employed to draw a non-linear frontier. Data from GBD database is utilized, and 500 bootstrap samples are used to calculate the average DALYs rate for each SDI value. The bootstrap method effectively assesses data uncertainty and variability. Local polynomial regression (LOESS) is used to smooth the frontier (11). Points on the frontier boundary indicate the theoretically achievable optimal health performance under given SDI conditions (12).

### 3.5 BAPC model projection

To forecast trends through 2035, a Bayesian age-period-cohort (BAPC) framework was implemented (13). This log-linear Poisson regression model assumes multiplicative interactions among age, period, and cohort effects on outcome variables, with each parameter modeled under Poisson distribution assumptions. The analysis utilized the BAPC package (v0.0.36) as an interface for the INLA package (v22.12.16), incorporating a framework-specific link function to execute Bayesian APC modeling via Integrated Nested Laplace Approximations within the R statistical environment.

All counts and rates are reported with 95% UI, which were generated by adopting the 2.5th and 97.5th percentiles obtained from 1,000 ordered draws of the posterior distribution. Within the Bayesian framework, uncertainty interval (also referred to as credible intervals) is constructed under the premise that there is a 95% probability that the true population value lies within this interval. All statistical analyses and data visualizations were performed using R (version 4.4.2) and JD_GBDR (V2.37, Jingding Medical Technology Co., Ltd.).

## 4 Results

### 4.1 Disease burden of decubitus ulcers

#### 4.1.1 Global trends

In 2021, the global incidence of DUs increased significantly from 1,142,594.78 cases in 1990 (95% UI: 1,030, 311.64–1,276, 015.91) to 2,468, 317.47 cases in 2021 (95% UI: 2,255, 077.26–2,720, 436.69). There was no significant difference in the rate of increase between males and females (Table 1; Supplementary Table S1). Similarly, prevalence rose from 300, 442.41 cases (95% UI: 270, 737.68–333, 578.99) in 1990 to 645, 588.11 cases (95% UI: 582, 431.80–712, 875.85) in 2021. Mortality demonstrated a substantial increase from 16,621.99 cases in 1990 (95% UI: 13, 738.32–19, 753.04) to 37, 032.73 cases 2021 (95% UI: 28, 523.11–42, 236.63). Over the 32-year period, the ASDR declined from 10 per 100,000 individuals in 1990 (95% UI: 8.87–12.74) to 9.70 per 100,000 individuals in 2021 (95% UI: 7.41–10.88).

**Table 1:** The number of disease burden cases of diarrheal disease in 2021, along with ASR and EAPC.

Characteristics	Incidence (95% uncertainty interval)			Prevalence (95% uncertainty interval)			Mortality (95% uncertainty interval)			DALYs (95% uncertainty interval)		
	Cases, 2021	ASIR, 2021	EAPC 1990–2021	Cases, 2021	ASPR, 2021	EAPC 1990–2021	Cases, 2021	ASMR, 2021	EAPC 1990–2021	Cases, 2021	ASDR, 2021	EAPC 1990–2021
Global	2468317.47 (2255077.26, 2720436.69)	30.28 (27.66, 33.27)	−0.02 (−0.10, 0.06)	645588.11 (582431.80, 712875.85)	7.92 (7.14, 8.73)	−0.02 (−0.11, 0.06)	37032.73 (28523.11, 42236.63)	0.46 (0.36, 0.52)	−0.58 (−0.71, −0.46)	803747.40 (612264.19, 903723.23)	9.70 (7.41, 10.88)	−0.58 (−0.78, −0.39)
SDI level												
High SDI	1147331.04 (1045411.07, 1267771.76)	54.09 (49.38, 59.61)	−0.17 (−0.22, −0.12)	304775.60 (273484.86, 336885.03)	14.41 (13.04, 15.83)	−0.18 (−0.23, −0.13)	6471.21 (5423.05, 7070.79)	0.26 (0.22, 0.28)	−2.47(−2.74, −2.20)	133708.95 (107391.54, 149671.25)	6.66 (5.87, 7.53)	−1.89 (−2.06, −1.72)
High-middle SDI	391169.19 (354133.45, 437425.87)	21.82 (19.81, 24.15)	0.31 (0.16, 0.46)	100826.70 (90594.11, 112013.57)	5.64 (5.09, 6.24)	0.28 (0.14, 0.43)	7763.52 (6165.05, 8671.36)	0.42 (0.33, 0.47)	2.09 (1.74, 2.45)	196155.65 (129297.26, 242116.97)	7.24 (5.80, 8.09)	1.59 (1.17, 2.00)
Low SDI	53230.92 (45658.36, 60688.05)	7.97 (7.08,8.95)	0.17 (0.07, 0.26)	12307.94 (10451.16, 14212.62)	1.59 (1.38, 1.79)	0.44 (0.29, 0.60)	3271.36 (2091.77, 4406.54)	0.77 (0.51, 1.03)	−0.50 (−0.65, −0.36)	237464.97 (173990.70, 269345.88)	16.45 (10.55, 22.02)	−0.88 (−1.12, −0.65)
Low-middle SDI	245510.59 (216421.74, 274059.25)	16.36 (14.74, 18.05)	1.06 (0.91, 1.21)	63031.36 (55740.93, 70472.90)	4.08 (3.69, 4.51)	1.16 (0.98, 1.34)	8098.22 (5287.20, 10112.61)	0.70 (0.46, 0.87)	−0.28 (−0.59, 0.03)	95957.95 (58607.26, 130148.10)	13.87 (9.21, 17.12)	−0.46 (−1.02, 0.11)
Middle SDI	628509.29 (569617.16, 693672.94)	26.29 (23.85, 28.86)	0.82 (0.68, 0.96)	163980.13 (148650.49, 180538.34)	6.83 (6.16, 7.52)	0.85 (0.70, 0.99)	11377.42 (8325.36, 13352.20)	0.53 (0.39, 0.62)	−0.18 (−0.24, −0.12)	139361.33 (123393.00, 157430.37)	9.80 (7.28, 11.15)	−0.23 (−0.34, −0.11)
GBD Region												
Andean Latin America	15340.44 (13818.29, 17075.85)	25.41 (22.87, 28.38)	−0.52 (−0.57, −0.47)	4071.07 (3651.40, 4506.38)	6.72 (6.02, 7.46)	−0.57 (−0.62, −0.51)	253.39 (197.64, 347.39)	0.46 (0.36, 0.63)	4.79 (4.17, 5.41)	4754.90 (3766.01, 6221.88)	8.19 (6.49, 10.69)	3.18 (2.31, 4.04)
Australasia	7895.09 (7047.03, 8943.00)	16.44 (14.58, 18.43)	−0.42 (−0.53, −0.31)	2092.96 (1862.59, 2357.35)	4.38 (3.91, 4.90)	−0.42 (−0.53, −0.31)	66.13 (53.34, 76.18)	0.10 (0.08, 0.11)	3.18 (2.40, 3.97)	1101.60 (934.69, 1262.79)	1.95 (1.65, 2.25)	2.57 (2.12, 3.02)
Caribbean	20585.08 (18804.87, 22624.41)	38.40 (35.12, 42.26)	−0.48 (−0.50, -0.46)	5395.97 (4899.80, 5927.07)	10.08 (9.13, 11.08)	−0.48 (−0.50, −0.46)	774.78 (657.32, 934.19)	1.41 (1.19, 1.71)	3.10 (2.57, 3.64)	15686.86 (12896.07, 19333.87)	29.72 (24.26, 36.76)	2.07 (1.44, 2.71)
Central Asia	3262.85 (2842.08, 3761.09)	4.46 (3.94, 5.13)	−0.01 (−0.05, 0.03)	860.53 (745.33, 994.37)	1.18 (1.03, 1.36)	0.00 (−0.04, 0.04)	17.05 (13.92, 21.06)	0.03 (0.02, 0.03)	1.97 (1.03, 2.93)	504.63 (418.53, 605.94)	0.66 (0.56, 0.79)	1.76 (1.40, 2.12)
Central Europe	110084.46 (99594.73, 123381.25)	50.66 (46.23, 56.17)	0.41 (0.33, 0.49)	28979.05 (25984.55, 32342.63)	13.38 (12.10, 14.86)	0.40 (0.32, 0.49)	245.63 (211.31, 276.23)	0.11 (0.09, 0.12)	1.28 (0.56, 2.01)	8530.66 (7041.05, 9967.00)	4.01 (3.29, 4.70)	1.62 (1.28, 1.95)
Central Latin America	200098.43 (182071.09, 219861.47)	82.05 (74.54, 90.36)	−0.24 (−0.28, −0.20)	53435.16 (48211.96, 58881.94)	21.89 (19.63, 24.19)	−0.25 (−0.29, −0.21)	1224.39 (1010.65, 1508.96)	0.52 (0.43, 0.64)	0.92 (0.78, 1.06)	29689.16 (24943.33,35831.22)	12.15 (10.23, 14.63)	1.03 (0.13, 1.95)
Central Sub-Saharan Africa	3972.97 (3376.75, 4640.28)	6.95 (6.27, 7.82)	−0.21 (−0.32, −0.10)	774.78 (628.26, 931.45)	0.94 (0.82, 1.08)	−0.38 (−0.41, −0.36)	506.21 (204.11, 791.97)	1.28 (0.52, 2.01)	0.71 (0.50, 0.91)	13971.28 (5085.87, 22087.45)	24.89 (10.26, 38.96)	0.84 (0.68, 0.99)
East Asia	404631.00 (366705.77, 452833.03)	22.20 (20.22, 24.58)	1.00 (0.78, 1.21)	104819.31 (94630.46, 116920.70)	5.76 (5.21, 6.37)	1.03 (0.81, 1.25)	3574.90 (1977.67, 4569.23)	0.22 (0.13, 0.29)	0.44 (0.39, 0.50)	68753.07 (42787.36, 84181.63)	3.88 (2.44, 4.74)	0.75 (0.55, 0.95)
Eastern Europe	69457.52 (61775.53, 79105.10)	22.74 (20.20, 25.59)	−0.37 (−0.46, −0.28)	18512.18 (16454.31, 21072.78)	6.08 (5.43, 6.79)	−0.37 (−0.46, −0.27)	416.10 (382.75, 449.14)	0.12 (0.11, 0.13)	0.06 (−0.13, 0.24)	11742.17 (10642.19, 13111.55)	3.78 (3.43, 4.24)	0.30 (0.24, 0.35)
Eastern Sub-Saharan Africa	12654.85 (10546.03, 15237.55)	7.90 (6.73, 9.35)	−0.54 (−0.63, −0.46)	1371.40 (1025.60, 1739.74)	0.47 (0.39, 0.56)	−0.52 (−0.67, −0.38)	2362.62 (1543.25, 3621.68)	1.66 (1.12, 2.49)	0.01 (−0.04, 0.06)	69405.67 (42324.96, 106586.37)	34.69 (22.73, 53.24)	0.16 (0.08, 0.25)
High-income Asia Pacific	196443.86 (177937.41, 219721.97)	47.65 (43.06, 52.64)	0.15 (0.06, 0.25)	52637.81 (47163.87, 59021.68)	12.89 (11.61, 14.17)	0.15 (0.06, 0.25)	1159.86 (912.52, 1339.86)	0.17 (0.13, 0.19)	−0.23 (−0.34, −0.13)	21934.44 (18767.59, 25475.07)	4.47 (3.74, 5.31)	0.02 (−0.25, 0.30)
High-income North America	766970.84 (696055.14, 843766.48)	115.45 (105.12, 126.72)	−0.05 (−0.15, 0.05)	204536.88 (182497.45, 225361.69)	30.82 (27.77, 33.94)	−0.08 (−0.19, 0.02)	1484.03 (1246.83, 1698.74)	0.21 (0.18, 0.24)	−0.27 (−0.57, 0.03)	54046.59 (44671.50, 63884.74)	8.28 (6.84, 9.80)	−0.46 (−0.65, −0.27)
North Africa and Middle East	57834.03 (50322.52, 64904.94)	11.42 (10.16, 12.73)	0.05 (−0.11, 0.20)	14909.71 (12860.98, 16858.11)	2.81 (2.49, 3.13)	−0.02(−0.19, 0.15)	2836.04 (2234.60, 3399.20)	0.83 (0.65, 0.99)	−0.38 (−0.45, −0.30)	62172.21 (49916.88, 73716.75)	14.57 (11.61, 17.23)	−0.81 (−0.91, −0.70)
Oceania	381.78 (335.24, 438.92)	5.85 (5.26, 6.52)	0.13 (0.10, 0.16)	78.21 (67.21, 91.05)	1.09 (0.98, 1.22)	0.09 (0.09, 0.10)	63.23 (23.15, 125.26)	1.08 (0.45, 2.02)	−1.20 (−1.91, −0.48)	1837.43 (621.00, 3785.34)	22.81 (8.61, 45.24)	−0.83 (−0.98, −0.69)
South Asia	212070.17 (181985.52, 241261.59)	12.04 (10.48, 13.55)	1.11 (0.86, 1.36)	55449.15 (47550.45, 63302.24)	3.05 (2.63, 3.46)	1.24 (0.98, 1.50)	6231.81 (3530.47, 8375.55)	0.52 (0.29, 0.70)	−1.28 (−1.39, −1.17)	162007.65 (100423.54, 214070.64)	10.91 (6.64, 14.34)	−0.93 (−1.23, −0.63)
Southeast Asia	44435.46 (40461.61, 49295.13)	8.22 (7.49, 9.01)	0.96 (0.84, 1.08)	8578.51 (7666.69, 9558.27)	1.45 (1.31, 1.60)	0.71 (0.62, 0.80)	7005.02 (4404.35, 8473.22)	1.42 (0.91, 1.72)	−1.94 (−2.58, −1.30)	137942.02 (85141.66, 168445.06)	23.85 (14.94, 28.79)	−1.17 (−1.26, -1.07)
Southern Latin America	17643.07 (16085.37, 19341.88)	19.86 (18.14, 21.77)	1.16 (0.90, 1.43)	4376.45 (3944.43, 4784.95)	4.95 (4.46, 5.40)	1.09 (0.83, 1.34)	1729.01 (1516.60, 1883.13)	1.87 (1.64, 2.03)	−2.02 (−2.40, −1.63)	25328.24 (22732.81, 27412.56)	28.05 (25.24, 30.33)	−1.33 (−2.53, -0.11)
Southern Sub-Saharan Africa	10717.53 (9468.91, 11911.13)	17.52 (15.84, 19.31)	−0.27 (−0.46, −0.08)	2616.64 (2315.99, 2928.31)	3.96 (3.55, 4.36)	−0.39(−0.53, −0.25)	778.92 (517.36, 952.00)	1.88 (1.25, 2.32)	−2.35 (−2.57, −2.12)	15461.82 (9815.01, 18909.37)	30.56 (20.09, 36.79)	−2.44 (−3.33, −1.54)
Tropical Latin America	178279.58 (160680.45, 196400.67)	72.25 (65.47, 79.35)	2.54 (2.23, 2.85)	47703.11 (43029.14, 52514.59)	19.33 (17.40, 21.20)	2.65 (2.33, 2.97)	1964.20 (1683.89, 2210.41)	0.81 (0.69, 0.91)	−2.53 (−2.75, −2.32)	41871.26 (37451.09, 46661.76)	16.84 (15.03, 18.78)	−2.59 (−2.76, −2.42)
Western Europe	125299.66 (113392.98, 140190.99)	13.29 (12.04, 14.79)	0.03 (−0.33, 0.39)	31702.24 (28483.34, 35337.66)	3.42 (3.09, 3.79)	0.19(−0.17, 0.56)	4283.73 (3475.84, 4727.06)	0.33 (0.27, 0.36)	−2.56 (−3.30, −1.81)	54444.84 (46295.77, 59470.72)	4.78 (4.14, 5.20)	−2.99 (−3.24, −2.73)
Western Sub-Saharan Africa	10258.79 (8356.59, 12299.41)	3.45 (2.97, 3.95)	0.18 (0.12, 0.25)	2686.98 (2159.43, 3245.85)	0.86 (0.73, 0.99)	0.11 (0.04, 0.18)	55.67 (10.84, 112.75)	0.03 (0.01, 0.07)	−3.14 (−3.69, −2.59)	2560.91 (886.75, 3986.50)	0.88 (0.31, 1.61)	−3.35 (−3.98, −2.71)

ASR, Age-standardized rates; ASIR, Age-standardized incidence rate; ASPR, Age-standardized prevalence rate; ASMR, Age-standardized mortality rate; ASDR, Age-standardized disability-adjusted life years rate; EAPC, Estimated annual percentage changes; DALYs, Disability-adjusted life years; SDI, SDI, Socio-demographic Index.

#### 4.1.2 SDI regional levels

**Figure 1:**
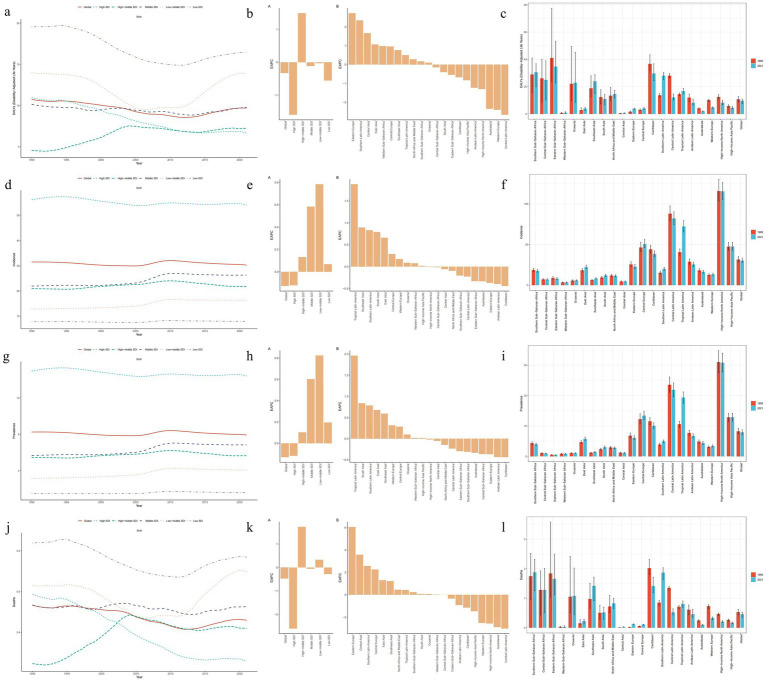
Changes in the burden of decubitus ulcers and a comparison between 1990 and 2021. **(a–c)** Age-standardized DALYs; **(d–f)** Age-standardized incidence; **(g–i)** Age-standardized prevalence; **(j–l)** Age-standardized mortality.

From the ASIR perspective, all SDI regions except high SDI areas exhibited increasing trends. High SDI regions demonstrated a decrease from 56.17 per 100,000 (95% UI: 50.83–63.12) to 54.09 per 100,000 (95% UI: 49.38–59.61), while middle SDI regions showed the most substantial increase (Table 1, Supplementary Table S1, and Figure 1). ASPR followed similar patterns, with only high SDI regions showing a marginal decline from 14.94 per 100,000 (95% UI: 13.37–16.68) to 14.41 per 100,000 (95% UI: 13.04–15.83). For ASMR, decreases occurred in high and low SDI regions, whereas middle SDI regions remained stable. Middle SDI regions recorded the highest EAPCs in both ASIR (0.82; 95% CI: 0.68–0.96) and ASPR (0.85; 95% CI: 0.70–0.99).

#### 4.1.3 Changes in 21 geographical regions

Substantial heterogeneity was observed in 2021 for ASIR, ASPR, ASMR, and ASDR of DUs across 21 GBD regions (Table 1; Supplementary Table S1). High-income North America, Central Latin America, and Tropical Latin America showed the highest growth trends in ASIR and ASPR. In terms of ASMR, Eastern Europe, Central Asia, and Southern Latin America had the highest ASMR, while Central Latin America, Australasia, and High-income Asia Pacific had the lowest. Additionally, Southern Sub-Saharan Africa, Eastern Sub-Saharan Africa, and the Caribbean were regions with relatively high ASDR. In the region of High-income North America, the ASIR and ASPR were the highest at 115.45 per 100,000 cases (95% UI: 105.12–126.72) and 30.82 per 100,000 cases (95% UI: 27.77–33.94), respectively. In the region of Southern Sub-Saharan Africa, the ASMR was the highest, at 1.88 per 100,000 cases (95% UI: 1.25–2.32). In the region of Western Sub-Saharan Africa, the ASMR and ASIR were the lowest at 0.03 per 100,000 (95% UI: 0.01–0.07) and 3.45 per 100,000 (95% UI: 2.97–3.95) respectively. In 2021, Central Asia had the lowest ASDR for certain conditions at 0.66 per 100,000 cases (95% UI: 0.56–0.79).

#### 4.1.4 National level

**Figure 2:**
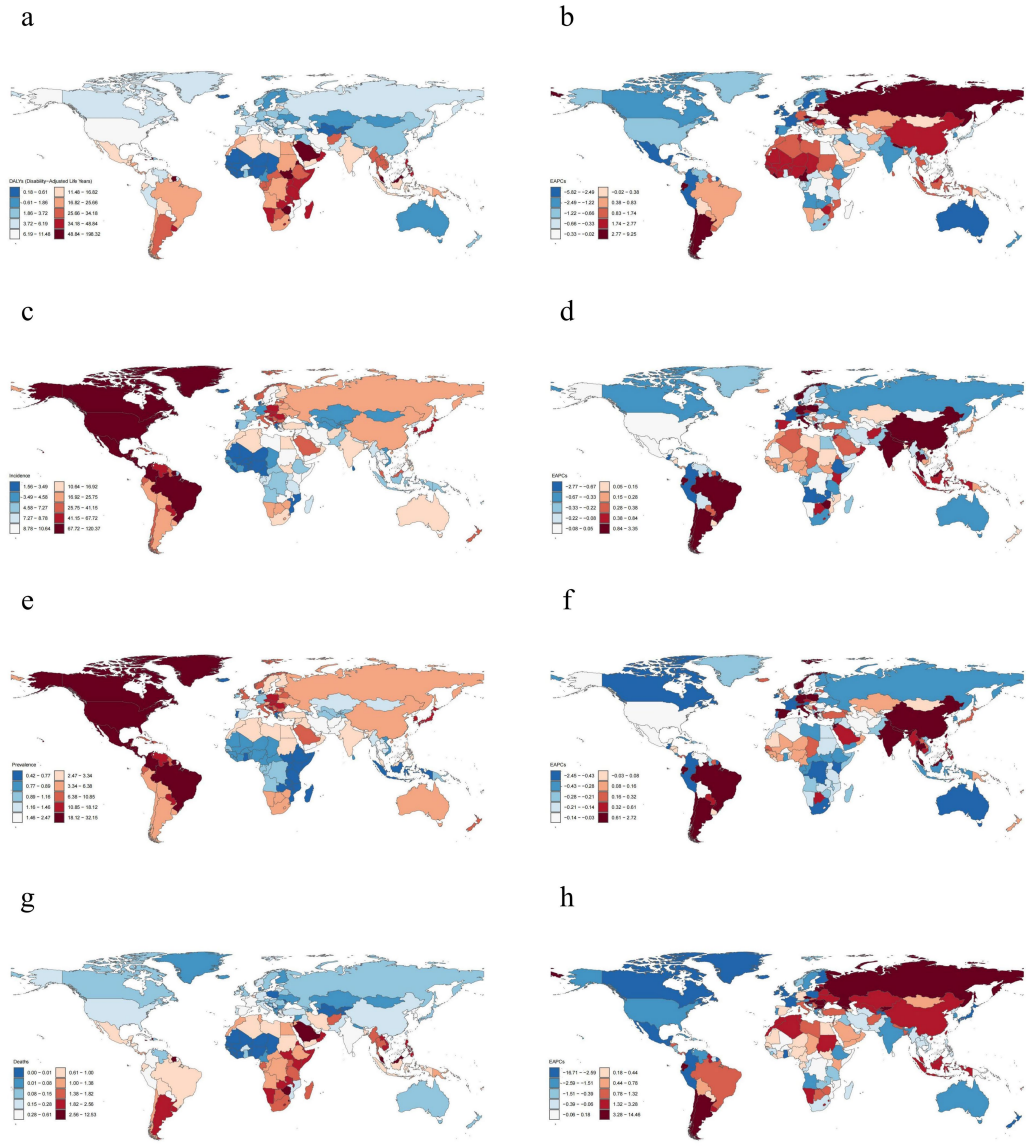
Global distribution of decubitus ulcers burden in 2021. **(a,b)** Age-standardized DALYs; **(c,d)** Age-standardized incidence; **(e,f)** Age-standardized prevalence; **(g,h)** Age-standardized mortality.

National-level analyses also integrated four key epidemiological metrics (Supplementary Table S2; Figure 2). In 2021, the United States of America recorded the highest incident (706, 150.43 cases; 95% UI: 640, 984.77–774, 801.46) and prevalent cases (188, 420.32 cases; 95% UI: 168, 469.47–207, 803.84). Meanwhile, India demonstrated the highest the highest mortality (5,441.37 deaths; 95% UI: 2, 999.48–7, 232.32) and disability burden (146, 565.01 DALYs; 95% UI: 90, 926.90–191, 409.94). After age-standardization, the United States of America was the highest ASIR and ASPR, while Barbados was the highest ASMR and ASDR. From the perspective of the EAPC, Georgia exhibited the fastest annual growth rates in mortality and DALYs. In terms of incidence and prevalence, Malaysia and Brazil demonstrated the fastest rates, respectively. Overall, the number of countries experiencing increases and decreases in these four indicators was roughly balanced.

### 4.2 Age and gender patterns

**Figure 3:**
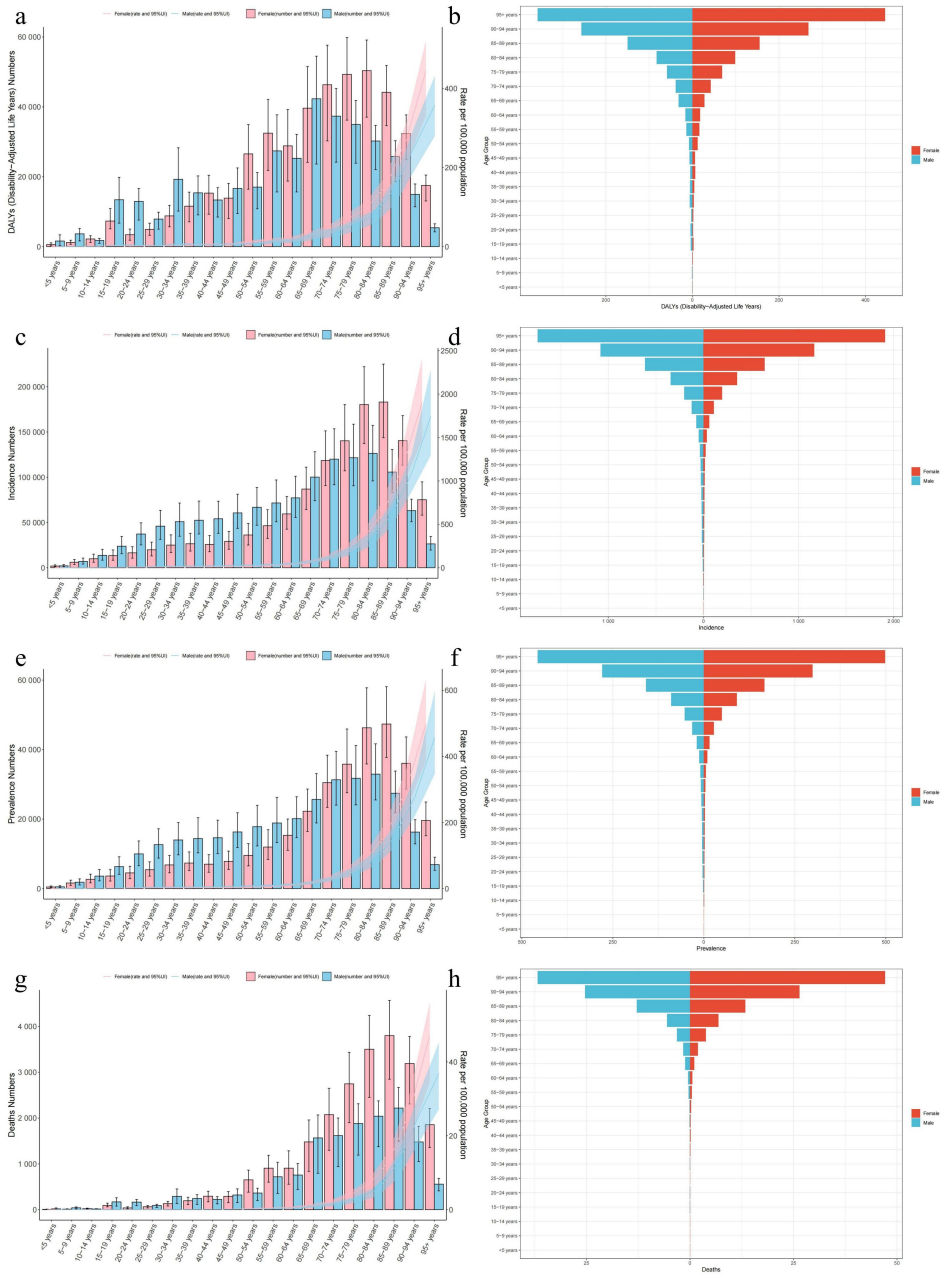
Distribution and trends (by age and sex) of the burden of decubitus ulcers in 2021. **(a,b)** DALYs; **(c,d)** Incidence; **(e,f)** Prevalence and **(g,h)** Mortality.

With advancing age, several epidemiological metrics exhibited distinct patterns of variation, including ASIR, ASPR, ASMR and ASDR (Figure 3). All these metrics demonstrated an overall increasing trend with age; however, their respective peaks occurred at disparate age intervals. Specifically, the peak ASDR among males diverged significantly from the other metrics, with a maximum observed between the ages of 65 and 69 years. In contrast, the peak ASDR among females occurred later, between the ages of 80 and 84 years. Notably, in the age group of 70 years and above, the ASDR for females were markedly higher than those for males. In 2019, ASIR case numbers reached their zenith in males aged 80–84 years and in females aged 85–89 years. Subsequently, a declining trend in case numbers was observed in both sexes. Below the age of 75 years, males generally exhibited higher ASIR case numbers compared to females. However, for individuals aged 75 years and above, the ASIR case numbers for females increased and surpassed those of males. The peak ASPR case numbers for both sexes aligned closely with the age ranges of incidence peaks, with slight variations observed between males and females. The age ranges at which ASPR case numbers increase for females were consistent with those for males. Lastly, ASMR case numbers for females were significantly higher than those for males, with peak values occurring between the ages of 85 and 89 years.

From the perspective of the 5 SDI regions and the 21 GBD regions, in terms of age patterns, incidence, prevalence, mortality, and DALYs in all regions were predominantly skewed towards individuals aged 80 and above, with a primary concentration in those aged and above (Supplementary Figure S1). In several regions—including Western Europe, Australasia, East Asia, Eastern Sub-Saharan Africa, and Central Sub-Saharan Africa—individuals aged ≥ 95 years accounted for >50% of decubitus ulcer-related mortality. Regarding the time patterns, in comparison to the data from 1990, Tropical Latin America witnessed the most significant increase in incidence and prevalence case numbers.

### 4.3 Join-point regression analysis

**Figure 4:**
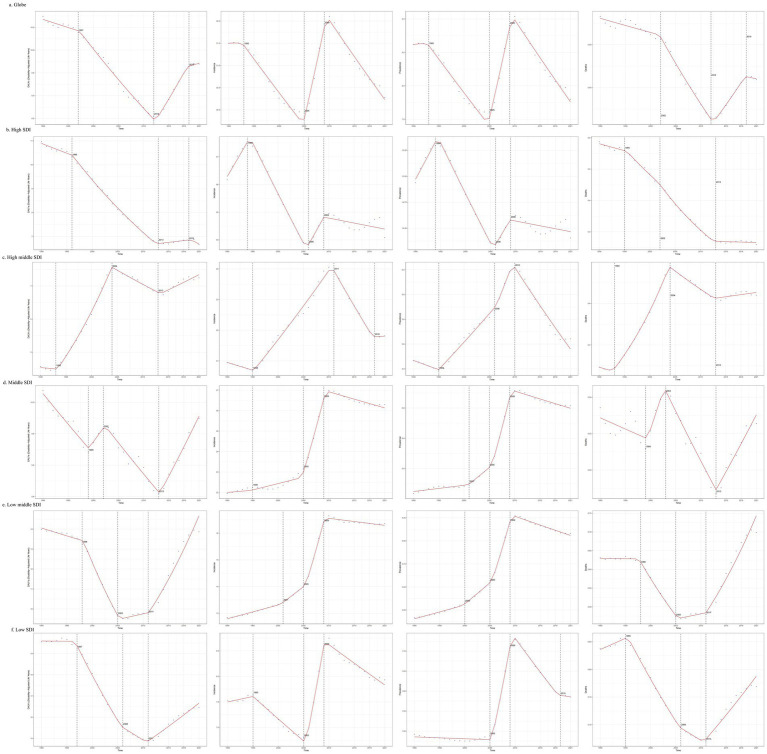
Join-point regression analysis of temporal trends in the burden of decubitus ulcers from 1990 to 2021.

Between 1990 and 2021, the global ASIR and ASPR of decubitus ulcers exhibited a cyclical pattern, initially declining, subsequently rising, and ultimately decreasing once more. The most significant increase occurred during the period of 2005–2009, with an APC of 1.700 (95%CI: 1.495–1.905; *p* < 0.001) for the incidence and an APC of 1.702 (95%CI: 1.491–1.913; *p* < 0.001) for the prevalence. The ASDR exhibited a pattern of initial decrease followed by an increase. ASMR trends showed non-significant declines post-2019 (*p* > 0.05), though concurrent significant surges occurred from 2012 to 2019 in both mortality (APC: 1.942; 95% CI: 1.569–2.317; *p* < 0.001) and DALYs (APC: 2.098; 95% CI: 1.777–2.420; *p* < 0.001), underscoring persistent concerns (Supplementary Table S3; Figure 4). The trend in ASIR and ASPR in high SDI regions followed a similar pattern to the global trend, but ASMR and ASDR exhibited a different trend. After 2012, there was a stabilizing trend rather than increase. In middle high SDI regions, ASMR and ASDR showed important turning points in 2004 and 2013, displaying cyclical variations, incidence and prevalence rates showed a similar turning point of increase in 1995. In middle SDI and low SDI regions, the trends in ASIR and ASPR were similar, with turning points of decrease in 2009. Both middle low SDI and low SDI regions showed the same turning point of increase in ASMR and ASDR, exhibiting an increasing trend after 2011 (Supplementary Table S3; Figure 4).

### 4.4 SDI correlations with region, country, and EAPC

**Figure 5:**
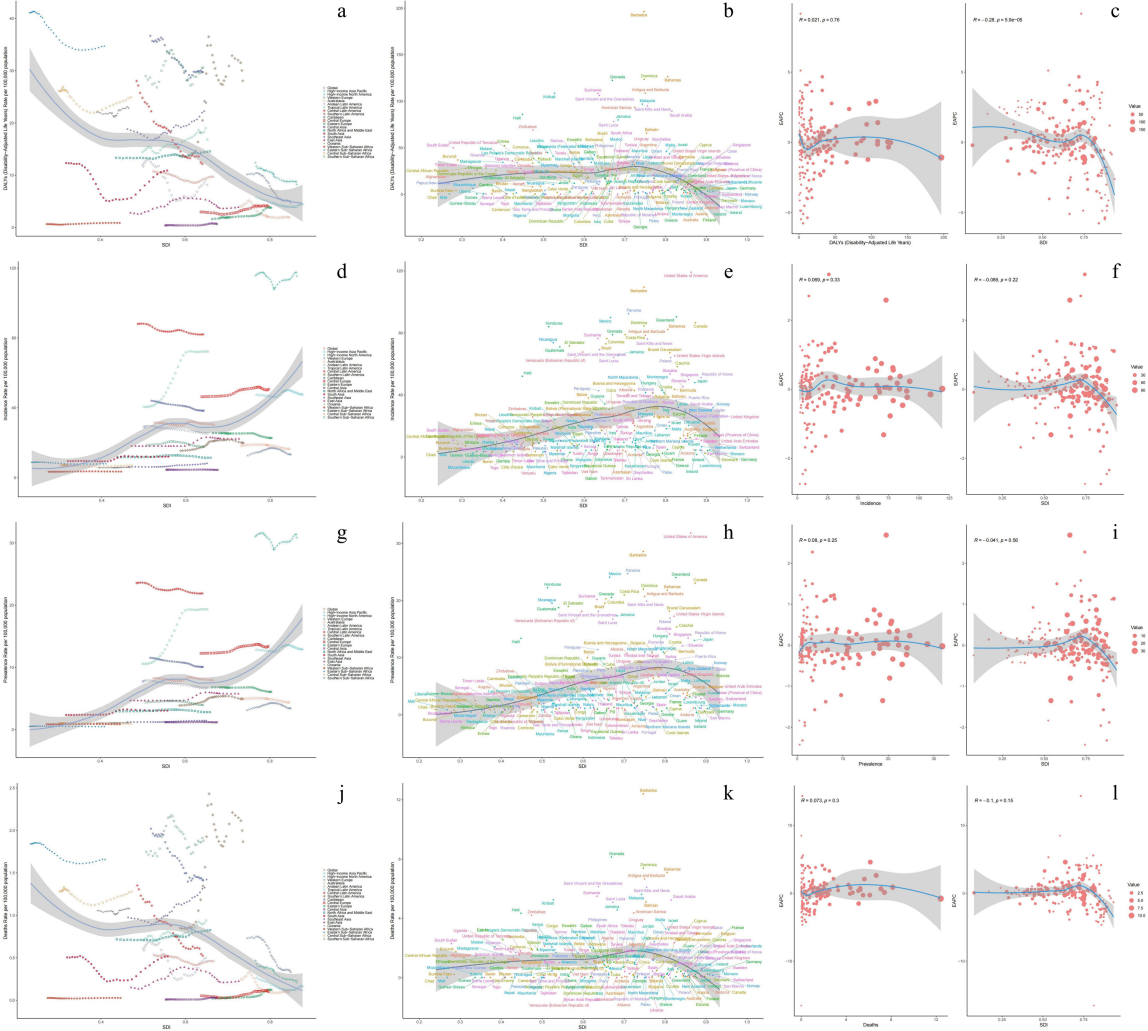
Correlation between ASR of decubitus ulcers and SDI at the national and regional levels in 2021. Correlation between estimated annual percentage change (EAPC) in 2021 and age-standardized rates of decubitus ulcers and SDI. In the left figures, circles represent countries, while in the right figures, circles represent countries for which human development index data is available. The size of each circle is proportional to the number of incidence, prevalence, mortality, and DALYs. **(a–c)** Age-standardized DALYs; **(d–f)** Age-standardized incidence; **(g–i)** Age-standardized prevalence; **(j–l)** Age-standardized mortality.

Further investigation was done on the 2021 SDI and the burden of decubitus ulcers in diverse regions and countries globally (Figure 5). On a regional level across 21 areas, ASIR, ASPR, ASMR, and ASDR all exhibited statistically significant correlations with SDI (*p* < 0.001). Notably ASDR (*R* = −0.4001, *p* < 0.001) and ASMR (*R* = −0.3706, *p* < 0.001) showed a negative correlation with SDI. ASIR (*R* = 0.5475, *p* < 0.001) and ASPR (*R* = 0.6077, *p* < 0.001) displayed a positive correlation with SDI, where the latter exhibited a more significant association. At the country level across 204 nations, ASIR (*R* = 0.3523, *p* < 0.001) and ASPR (*R* = 0.4840, *p* < 0.001) remained significantly correlated with SDI. However, ASDR (*R* = −0.1546, *p* = 2.e-02) and ASMR (*R* = −0.1218, *p* = 8.279e-02) showed no statistically correlation with SDI. In subsequent analysis introducing EAPC, only a negative correlation was observed between SDI and ASDR (*R* = −0.28, *p* = 5.9e-05).

### 4.5 Health inequality analysis

**Figure 6:**
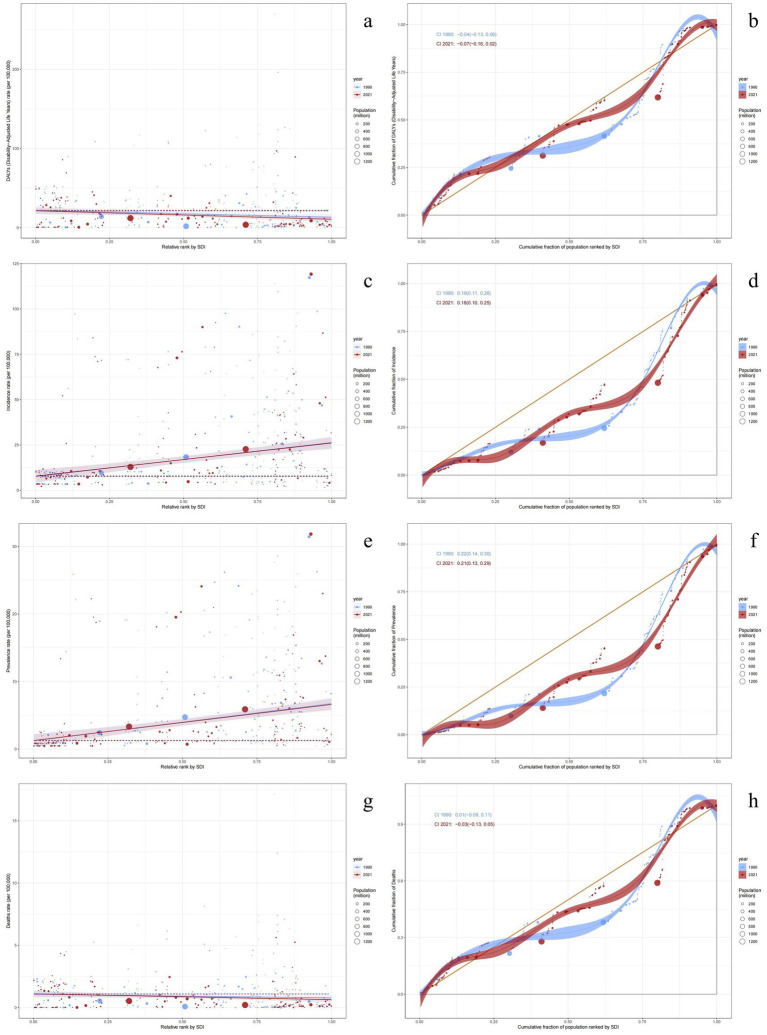
SDI-related health inequality regression curves and concentration curves for the global burden of decubitus ulcers, 1990 and 2021. The health inequality regression curve on the left and the concentration curve on the right. **(a,b)** Age-standardized DALYs; **(c,d)** Age-standardized incidence; **(e,f)** Age-standardized prevalence; **(g,h)** Age-standardized mortality.

The relationship between the SDI and health inequality was assessed using the SII and CI (Supplementary Table S4; Figure 6). In 2021, SII values indicated that both ASIR and ASPR increased with higher socioeconomic status, with ASIR exhibiting more pronounced inequality. Compared to 1990, ASIR and ASPR trends reflected marginally improved equity (evidenced by slight SII decreases), though changes were minimal. In contrast, the ASMR and ASDR consistently exhibited negative correlations with socioeconomic status, and this inequality has been widening over time. Notably, ASDR shifted from −8.89 (95% CI: −16.42 to −1.36) in 1990 to −10.73 (95% CI: −17.30 to −4.15) in 2021.

Notably, the CI for ASMR transitioned from positive to negative between 1990 and 2021, indicating a shift from positive correlation with SDI (higher burden in advantaged groups) to negative correlation (higher burden in disadvantaged groups). This pattern aligned with ASDR trends in 2021. ASIR and ASPR exhibited no significant CI changes.

### 4.6 Decomposition analysis

Decomposition analysis was employed to further explore the contributions of different factors to the disease burden (Supplementary Figure S2). From 1990 to 2021, the increase in global DUs incidence was primarily driven by population growth (51.02%) and aging (53.34%), while epidemiological changes (−4.35%) slowed the rise in incidence, partially offsetting this growth. The remaining indicators showed a similar proportionate impact on incidence. Except for high SDI regions, population growth and aging were the main driving forces for all evaluation indicators in the other four SDI regions. Notably, in high SDI regions, the increase in mortality was influenced by population growth (1,609.47%) and aging (1,979.75%), while epidemiological changes (−3,489.22%) were the main factor in reducing mortality. The DALYs trend in this region also showed a significant proportionate impact, driven by population growth (208.95%) and aging (253.18%), with epidemiological changes acting as a reducing factor (−362.13%). Among the 21 regions, South Asia exhibited significant differences in DALYs compared to other regions, with aging and epidemiological changes being the dominant factors, and less influence from population growth. Moreover, population aging was the main reducing factor in this region.

### 4.7 Frontier analysis

Frontier analysis of ASIR, ASPR, ASMR, and ASDR was conducted to understand the potential improvement space for the burden of ulcers in different countries based on their SDI. This analysis aims to identify opportunities for enhancing the burden of pressure ulcers based on the SDI of each country (Supplementary Figure S3).

In terms of mortality rates and DALYs, countries in the high-middle SDI regions exhibited significant disease burdens. While Barbados showed a declining trend, it consistently remained at the forefront of the burden. Other regions, such as Grenada, Dominica, and the Bahamas, exhibited upward trends following Barbados. Regarding incidence and prevalence rates, the United States of America (high SDI region) consistently remained at the forefront of the disease burden with an upward trend, closely followed by Barbados and Panama (high middle SDI regions). Barbados demonstrated an increasing trend in incidence rate but a decreasing trend in prevalence rate. Overall, the highest disease burden was observed primarily in high-middle SDI regions.

### 4.8 Forecasting trends in the burden of DUs

This study conducted future projections and trend analyses for the ASIR, ASPR, ASMR, and ASDR of DUs (Supplementary Figure S4). By 2035, the ASIR for DUs is estimated to be 30.92 per 100,000 population (95%UI: 28.79–33.05). ASPR is expected to be 8.08 per 100,000 population (95%UI: 7.49–8.66). ASMR is forecasted to be 0.47 per 100,000 population (95%UI: 0.39–0.56). ASDR is estimated to be 9.48 per 100,000 population (95%UI: 7.86–11.09).

Despite an anticipated overall decline in the disease burden of DUs, a rising trend in certain age cohorts warrant attention. More precisely, ASMR and ASDR demonstrated an increasing trend in certain age groups, including those aged 50 to 54 years, 55 to 59 years, and 75 to 79 years, though the magnitude of increase was relatively limited. Notably, ASIR and ASPR showed a pronounced upward trend among individuals aged 85 to 89 and 90 to 94 years, highlighting persistent challenges in the geriatric population. Additionally, a mild increase is also observed in the age groups of 40 to 44, 45 to 49, and 50 to 54 years, which also merit attention.

## 5 Discussion

### 5.1 Overview of study findings

This study provides a comprehensive analysis of the global burden of DUs from 1990 to 2021, integrating epidemiological trends, socioeconomic determinants, and age-gender disparities. Key findings included a significant rise in global incidence and prevalence of DUs, driven by population growth and aging, while mortality and DALYs showed modest declines. From the perspective of the SDI levels, high SDI regions demonstrated relatively effective prevention and control of DUs. The ASIR, ASPR, ASMR, and ASDR had all shown varying degrees of decline in these regions. Notably, in terms of ASIR and ASPR, high SDI regions were the exception, as other regions had generally experienced increases in these indicators. Middle SDI regions experienced the largest increase in these indicators. Gender disparities persisted, with older females facing higher mortality and DALYs. Socioeconomic development, as measured by the SDI, exhibits a complex relationship with DUs. Higher SDI correlated with elevated incidence and prevalence but lower mortality and DALYs. Health inequality still exists. There was a trend of increasing in terms of DALYs. Aging and population growth were the main factors driving the increase in the disease burden of DUs. The United States of America and Barbados were at the forefront of the global disease burden. Although the global disease burden forecasts for DUs showed a downward trend, an upward trend existed among those aged 40–54, which suggested that in the future, there may be a trend of earlier onset of DUs. This study contributes valuable information that could inform public health interventions and clinical practices in reducing the burden of DUs worldwide.

### 5.2 Comparison with previous studies

Although a cursory examination revealed that the global incidence, prevalence, mortality, and DALYs of DUs are on the rise, these four epidemiological indicators demonstrated a downward trend after age standardization. This further underscores the significant role of age structure in the disease burden of pressure ulcers. This finding is in concordance with the results from the decomposition analysis. These are also consistent with previous research results, indicating that aging are key drivers of the burden of DUs (14, 15).

Consistent with previous research, as economic development proceeds and the performance of healthcare systems in various countries improves, the ASMR and ASDR of DUs are also declining (16). However, through comprehensive comparisons, inequality analyses, and frontier analyses, it is evident that although high SDI regions have well-developed healthcare systems, factors such as population aging (17), high hospitalization rates (18, 19), and certain dietary habits (20) significantly increased the disease burden in these areas.

As a developed country, the United States has a relatively sound medical infrastructure. However, a high number of hospitalizations may also be an important reason for the high incidence of DUs in the country. The United States had the highest incidence rate globally. And even after age adjustment, it still ranked first. Although comprehensive medical equipment had significantly reduced the mortality rate and DALYs of the disease, it had also created a substantial economic burden (17).

India had the highest number of mortality and DALYs. The increasing number of diabetic patients may be an important reason for the rise in mortality from DUs in India. Multiple studies have shown that a significant proportion of diabetic patients in India die from DUs (21, 22). However, the impact of India’s age structure on mortality rates and DALYs cannot be ignored. Therefore, it is essential to conduct comparisons after age-standardization. After age standardization, the ASMR and ASDR become more convincing. Following age-standardization, Barbados ranks first. The highest ASDR and ASMR have exacerbated the disease burden in Barbados. This is also consistent with the results of the frontier analysis.

Join-point regression analysis revealed that after 2005, there was a linear upward trend in incidence and prevalence. This may be associated with the avian influenza outbreak that occurred globally in 2005, mainly involving the H5N1 virus (23). The outbreak emerged in several countries and regions, drawing global attention and concern. The increase in hospitalization rates may had contributed to the elevated prevalence and incidence of DUs. After 2009, the incidence and prevalence showed a downward trend. It was in this year that some countries introduced relevant policies to further strengthen the management of DUs. In particular, the National Institute for Health and Care Excellence in the UK released the guideline “Prevention and Management of Pressure Ulcers in Primary and Secondary Care.” The study by Anders J et al. also found that pressure ulcers in bedridden patients have become less common (24). It was also in this year that the high incidence and prevalence of DUs attracted significant attention (25). The ASMR and ASDR had been on a continuous downward trend, but a turning point occurred in 2012 when both rates began to rise. Coincidentally, it was in 2012 that a novel coronavirus was discovered, later named the Middle East respiratory syndrome coronavirus. Moreover, 2012 marked a phase of early research and surveillance for the Zika virus. The high hospitalization rates and the high mortality rates of these viruses could be significant factors contributing to the emergence of this turning point.

### 5.3 Potential explanations for observed trends and clinical significance

The escalating global burden of DUs is a multifaceted issue, intricately linked to demographic, socioeconomic, and epidemiological dynamics. This study aims to elucidate the underlying factors contributing to this phenomenon and highlight potential areas for intervention.

#### 5.3.1 Demographic shifts: population growth, aging

The significant increase in DUs incidence, particularly in middle SDI regions, is largely attributable to demographic shifts, with population growth and aging accounting for 51–53% of the incidence rise. Aging is a crucial factor, as it amplifies frailty, immobility, and comorbidities, thereby directly escalating the risk of DUs (26, 27). As life expectancy rises, the prevalence of age-related conditions such as sarcopenia and impaired circulation further complicates DUs prevention and management (28, 29). Preventative measures for DUs primarily include skin assessment, appropriate positioning and repositioning, the use of pressure-redistributing devices, and the application of prophylactic dressings (30). Regular assessment of skin integrity, including color, temperature, firmness, and moisture levels, is also crucial for the timely identification of DUs risk in the older adult population (31). Furthermore, targeted surveillance in densely populated areas, particularly those with high hospital admission rates, is an important measure for effectively controlling the disease burden of DUs.

#### 5.3.2 Socioeconomic dynamics: disparities in healthcare and prevention

The SDI play a pivotal role in shaping the epidemiology of DUs. High SDI regions have witnessed a decline in ASMR and ASDR of DUs, which can be attributed to improved preventive care, including the adoption of advanced pressure-relief technologies (32, 33) and early diagnostic measures (34–36). These advancements reflect the positive impact of socioeconomic development on healthcare infrastructure and accessibility. The negative correlation between SDI and DUs mortality underscores the importance of advanced wound care and rehabilitation in reducing fatalities, particularly in wealthier nations. Middle SDI regions, facing dual challenges of limited healthcare resources and aging populations, require urgent investment in evidence-based preventive measures and specialized wound care services to bridge this gap (37). Healthcare authorities should develop DUs management strategies tailored to local epidemiological characteristics. Specifically, high SDI regions should prioritize DUs prevention. Medium SDI regions should focus on control in densely populated areas and efficient resource allocation. Low SDI regions should concentrate on tailored interventions to reduce mortality.

#### 5.3.3 Gender and age-specific vulnerabilities: biological and social determinants

Gender and age-specific vulnerabilities further complicate the epidemiology of DUs. Higher mortality rates among older females are attributed to a combination of biological and social factors. Biologically, women are more susceptible to DUs due to thinner skin and a higher prevalence of osteoporosis, which compromise tissue integrity and increase the risk of DUs (38). Socially, gender disparities in caregiving responsibilities and longer periods of institutionalization among older adult women exacerbate their vulnerability (39). The projected rise in ASIR and ASPR among the oldest-old (85–94 years) highlights systemic inadequacies in geriatric care globally. This underscores the need for gender-sensitive and age-specific strategies to address the unique challenges faced by older adult women and improve overall geriatric care standards. For high-risk individuals, prophylactic dressings may be applied to bony prominences (such as the sacrococcygeal region, heels, and occiput) and medical device contact sites, with foam and hydrocolloid dressings being the most commonly used (40).

### 5.4 Strengths and limitations

This study offers several strengths that contribute to its robustness and relevance. Firstly, it leverages the GBD 2021 datasets, which is renowned for its rigorous methodology, including Bayesian hierarchical models and spatiotemporal adjustments (41). These advanced techniques enhance the comparability of findings across different regions, providing a comprehensive and reliable basis for analysis. Secondly, the multidimensional approach, incorporating the SDI, age-gender stratification, and decomposition analysis, allows for nuanced insights into the drivers of the disease burden of DUs. This multifaceted methodology provides a deeper understanding of the underlying factors contributing to the burden. Thirdly, the use of the BAPC model for forecasting offers actionable predictions that can guide policymakers in developing targeted interventions and resource allocation strategies (13). Additionally, inequality analysis, decomposition analysis, and frontier analysis further dissect the global situation of the disease burden of DUs, significantly enhancing the credibility and comprehensiveness of the conclusions.

Despite these strengths, several limitations must be acknowledged. Firstly, the reliance on secondary data may introduce biases, particularly in low SDI regions where underreporting of DU cases is common due to limited diagnostic infrastructure. This limitation may affect the accuracy and completeness of the data, potentially skewing the results. Secondly, the ecological study design precludes causal inferences between SDI and DUs trends. Unmeasured confounders, such as cultural care practices and staffing ratios in nursing homes, may also influence outcomes, further complicating the interpretation of the results. Thirdly, the predictions of BAPC models are typically based on statistical patterns derived from historical data, assuming future trends will follow past patterns. However, they may neglect unpredictable factors such as public health emergencies, policy interventions, or technological breakthroughs. Moreover, while BAPC models rely on historical associations of fixed risk factors for projections, they fail to dynamically integrate the evolution of future risk factors like emerging environmental pollutants or lifestyle changes. Lastly, due to limitations in the volume of GBD data, we were unable to analyze the population characteristics of DUs across different stages (e.g., stage I - IV). Consequently, this study could not provide a more comprehensive description of the demographic features associated with each DUs stage. This limitation impacts the applicability of our findings for guiding regional healthcare policy formulation, particularly given that clinical management strategies differ significantly across DUs stages. Similarly, this study was unable to perform subgroup analyses comparing hospital-acquired versus community-acquired cases, nor on different types of high-risk populations (e.g., spinal cord injury, post-operative). To monitor the dynamic changes in the burden of decubitus ulcers, it is essential to improve the quality and granularity of health data on decubitus ulcers in all regions and countries.

## 6 Conclusion

In summary, DUs are a global public health issue, with significant differences existing among different regions and countries. The burden of DUs is most pronounced in older adult patients. Prioritizing preventive strategies (e.g., caregiver training, pressure-relief devices) and equitable resource allocation could mitigate future burdens. Further research should explore localized interventions and the impact of healthcare policies on DUs outcomes.

## Data Availability

The original contributions presented in the study are included in the article/Supplementary material, further inquiries can be directed to the corresponding authors.

## Footnotes

^1^ http://ghdx.healthdata.org/gbd-results-tool

## Supplementary material

SUPPLEMENTARY FIGURE S1 Age distribution characteristics for five SDI regions and 21 areas. **(a,b)** Age-standardized DALYs; **(c,d)** Age-standardized incidence; **(e,f)** Age-standardized prevalence; **(g,h)** Age-standardized mortality.

SUPPLEMENTARY FIGURE S2 Key drivers of decubitus ulcers burden at global, SDI levels, 21 regions from 1990 to 2021: population growth, ageing, and epidemiological changes. The black dots represent the sum of contributions to changes in all three factors. **(a,b)** Age-standardized DALYs; **(c,d)** Age-standardized incidence; **(e,f)** Age-standardized prevalence; **(g,h)** Age-standardized mortality.

SUPPLEMENTARY FIGURE S3 Frontier analysis involving SDI and decubitus ulcers burden in 2021. **(a)** Age-standardized DALYs; **(b)** Age-standardized incidence; **(c)** Age-standardized prevalence; **(d)** Age-standardized mortality.

SUPPLEMENTARY FIGURE S4 Trends in the burden of decubitus ulcers (overall and by age group): Observed rates (1990-2021) and predicted rates (2022-2035). **(a,b)** Age-standardized DALYs; **(c,d)** Age-standardized incidence; **(e,f)** Age-standardized prevalence; **(g,h)** Age-standardized mortality.
