# Supplementary figures and images for: Global, regional, and national burden of decubitus ulcers, 1990–2021: analysis of the current situation, multidimensional analysis, and trend forecasting for the global burden of disease study 2021
Source: Front Med (Lausanne). 2025 Jul 2;12:1588032. doi: 10.3389/fmed.2025.1588032 (PMC12263942; doi:10.3389/fmed.2025.1588032)

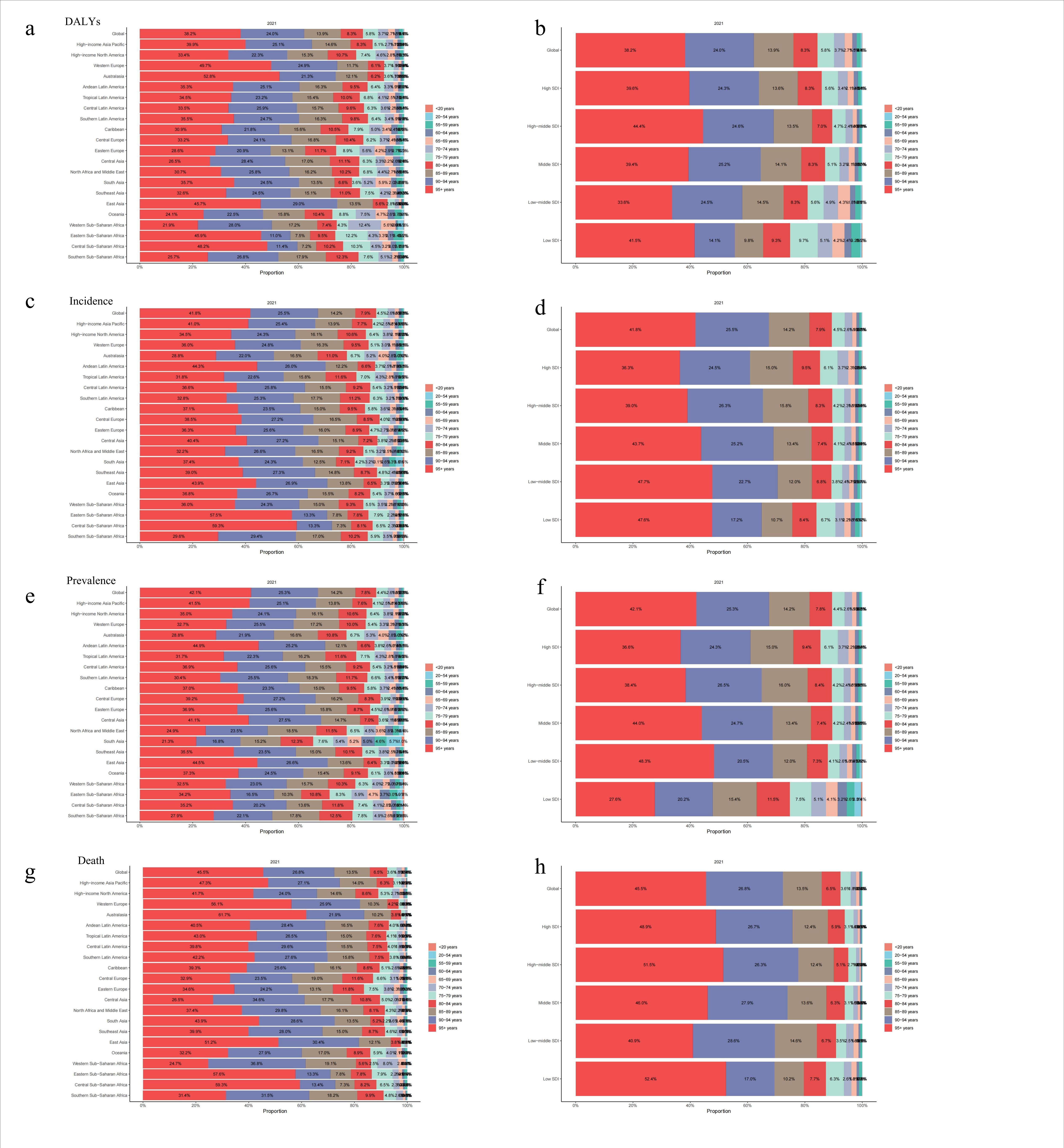

Supplement: SUPPLEMENTARY FIGURE S1 — Age distribution characteristics for five SDI regions and 21 areas. (a,b) Age-standardized DALYs; (c,d) Age-standardized incidence; (e,f) Age-standardized prevalence; (g,h) Age-standardized mortality. [file Image_1.jpeg]

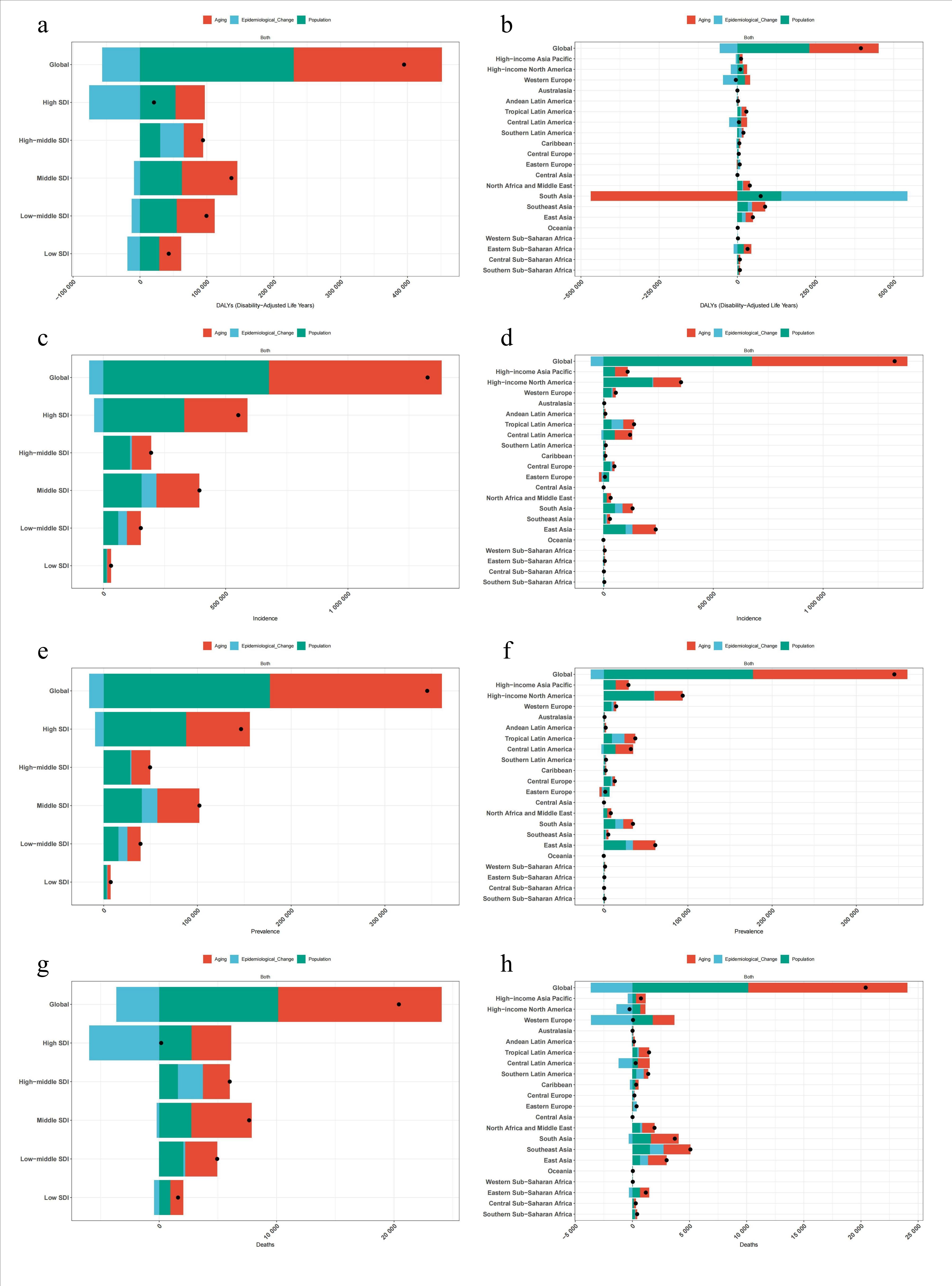

Supplement: SUPPLEMENTARY FIGURE S2 — Key drivers of decubitus ulcers burden at global, SDI levels, 21 regions from 1990 to 2021: population growth, ageing, and epidemiological changes. The black dots represent the sum of contributions to changes in all three factors. (a,b) Age-standardized DALYs; (c,d) Age-standardized incidence; (e,f) Age-standardized prevalence; (g,h) Age-standardized mortality. [file Image_2.jpeg]

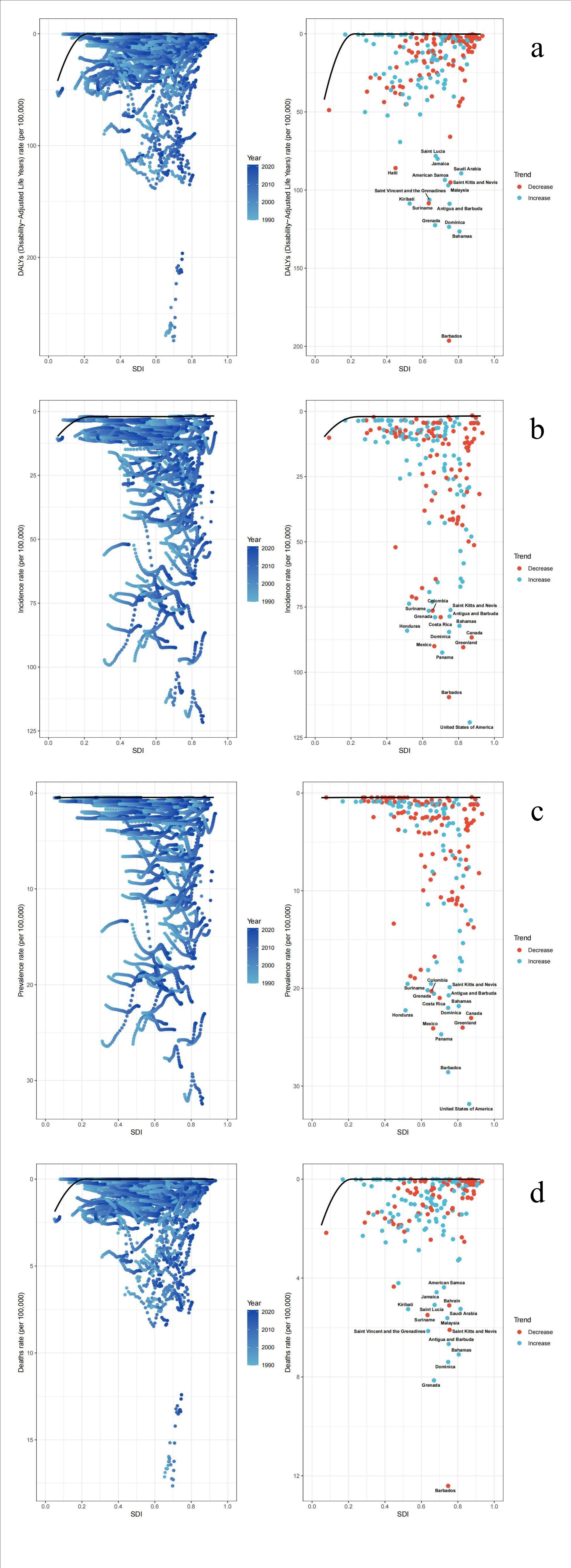

Supplement: SUPPLEMENTARY FIGURE S3 — Frontier analysis involving SDI and decubitus ulcers burden in 2021. (a) Age-standardized DALYs; (b) Age-standardized incidence; (c) Age-standardized prevalence; (d) Age-standardized mortality. [file Image_3.jpeg]

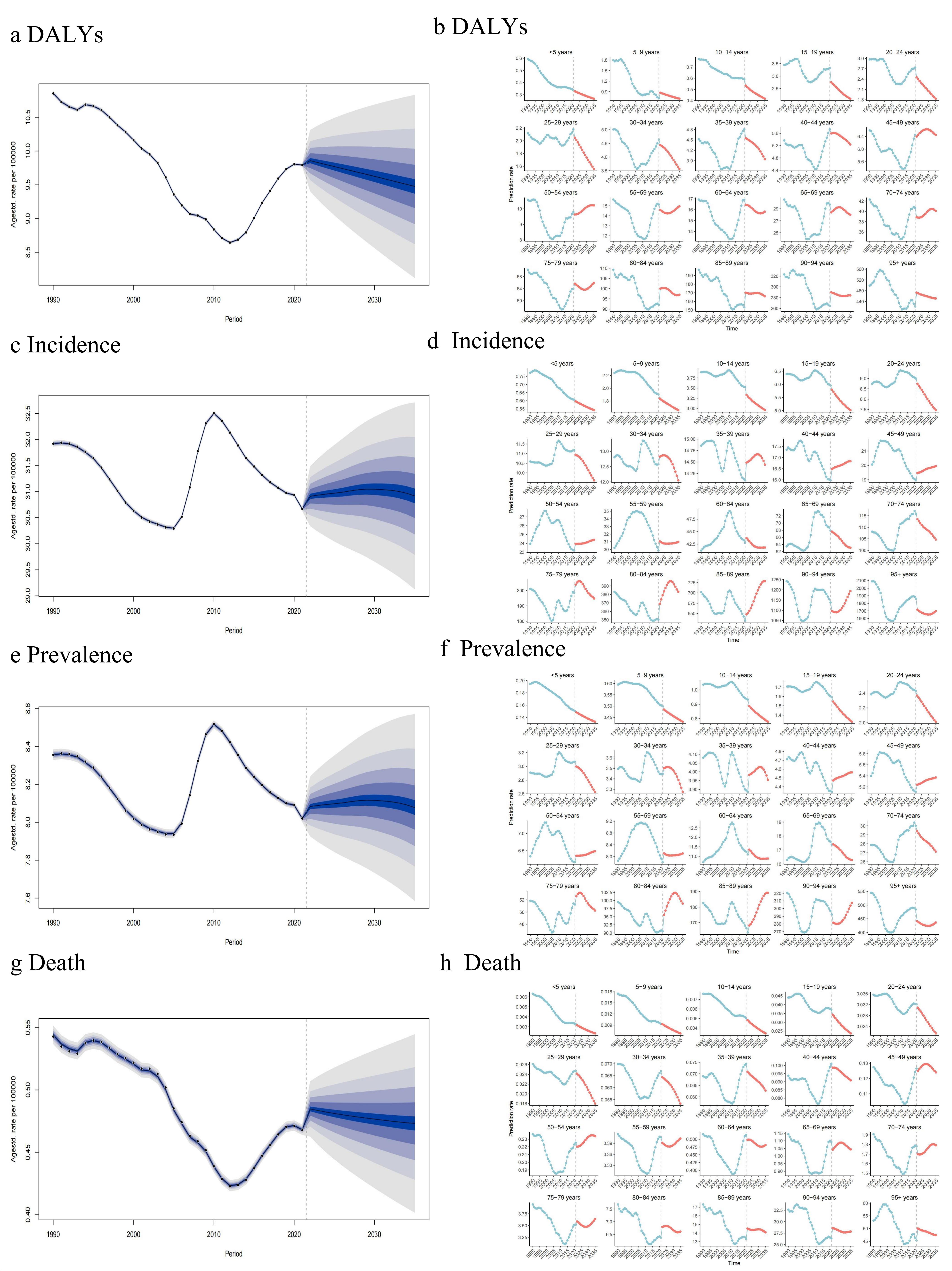

Supplement: SUPPLEMENTARY FIGURE S4 — Trends in the burden of decubitus ulcers (overall and by age group): Observed rates (1990-2021) and predicted rates (2022-2035). (a,b) Age-standardized DALYs; (c,d) Age-standardized incidence; (e,f) Age-standardized prevalence; (g,h) Age-standardized mortality. [file Image_4.jpeg]
